# The potential immuno-stimulating effect of curcumin, piperine, and taurine combination in hepatocellular carcinoma; a pilot study

**DOI:** 10.1007/s12672-023-00785-1

**Published:** 2023-09-13

**Authors:** Raghda R. kotb, Ahmed M. Afifi, Motawa E. EL-Houseini, Mohamed Ezz-Elarab, Emad B. Basalious, Mervat M. Omran, Mona S. Abdellateif

**Affiliations:** 1 Department of Zoology, Faculty of Science, Cairo University, Cairo, Egypt; 2 Medical Biochemistry and molecular biology, Cancer Biology Department, National Cancer Institute, Cairo University, Cairo, Egypt; 3 National Hepatology and Tropical Medicine Research Institute, Cairo, Egypt; 4 Pharmaceutics and Industrial Pharmacy department, Faculty of Pharmacy, Cairo University, Cairo, 11562 Egypt; 5 Pharmacology Unit, Cancer Biology Department, National Cancer Institute, Cairo University, Cairo, Egypt; 6 Biological science division, University of Chicago, Chicago, IL USA

**Keywords:** Liver cancer, HCC, Curcumin, Immunomodulation, Piperine, Taurine

## Abstract

**Background:**

This is a phase II clinical trial to investigate the immunotherapeutic effect of Curcumin, Piperine, and Taurine (CPT) combination in hepatocellular carcinoma (HCC).

**Methods:**

Twenty-six HCC patients aged (50–80 years) were recruited for administration of a daily dose of 5 g of curcumin, 50 mg of piperine, and 500 mg of taurine divided into three doses for successive 3 months. The three components (CPT) were prepared in one capsule. Patients were assessed after each month (cycle) for the plasma levels of CD4, CD8, CD25, Interleukins-2 (IL-2), IL-6, IL-12, Interferon-gamma (IFN- γ), Lactate dehydrogenase (LDH), and Vascular endothelial growth factor (VEGF), *FOXP3* mRNA, and *miRNA 21*.

**Results:**

There was a significant increase in the plasma levels of CD4 and CD8, while a significant decrease in the CD25 level after the second and third cycles compared to the baseline level [P < 0.001 for both]. Also, there was a significant increase in the plasma levels of IL-2, IL-12, and IFN-γ [ P = 0.001, P = 0.006, and P = 0.029; respectively], while there was a significant decrease in IL-6, VEGF-α, LDH, and Alpha-fetoprotein (AFP) after CPT administration compared to the baseline levels [P < 0.001, P < 0.001, P = 0.020, and P = 0.004; respectively].

The expression level of *miRNA-21* was significantly decreased after CPT administration compared to the baseline level [5.5±0.88, 4.1±0.78, 3±0.75, and 2.5±0.76; respectively, P<0.001]. Though there was a noticeable decrease in the *FOXP3* expression after each cycle, however, it didn’t reach a significant level [5.3±0.8, 4.2±0.76, 3.2±0.67, and 2.5±0.79; respectively, P=0.184].

**Conclusion:**

CPT could exhibit a potential immune-stimulating effect in HCC patients. The current trial had been registered at the National Hepatology and Tropical Medicine Research Institute (NHTMRI), with a registration number of NHTMRI-IRB 2-21 on 5th January 2021.

**Supplementary Information:**

The online version contains supplementary material available at 10.1007/s12672-023-00785-1.

## Introduction

Hepatocellular carcinoma (HCC) is a major public health problem, it represents the sixth most diagnosed cancer, and the third most lethal cancer worldwide [1]. It has an increasing incidence of morbidity and mortality in both developing and developed countries [2]. HCC is a heterogeneous disease with various risk factors, where hepatitis B (HBV) and aflatoxin exposure are considered the main risk factors for HCC in Africa, while hepatitis C (HCV) is the major risk factor for HCC in USA, Europe, and Japan [3, 4].

Indeed, the clinical outcome of HCC patients is usually unfavorable, where the surgical option is suitable only for early-stage patients who represent about 5–15% of all HCC cases. However, the risk of postoperative complications is commonly experienced due to decreased hepatic regenerative capacity. While intermediate-stage liver cancer patients are suitable candidates for trans-arterial chemoembolization (TACE) therapy, which achieves only a 23% improvement in 2-year survival rate [5]. Additionally, there are many molecularly targeted drugs that have been approved by the WHO for that treatment of patients with advanced disease. These drugs include kinase inhibitors (e.g. sorafenib, regorafenib, Lenvatinib, and cabozantinib), angiogenesis inhibitors (e.g. ramucirumab and bevacizumab), and immune checkpoint inhibitors (e.g. pembrolizumab, atezolizumab, and nivolumab). However, the survival rate of HCC patients is very low and extends only for a few months due to the emergence of secondary resistance and drug toxicity [6, 7]. Liver transplantation is an optimal choice for HCC patients, however lacking donors, high cost, and the complications of immunosuppressive drugs make it of limited application [8, 9]. Taken together, HCC is considered a lethal cancer, where about 70% of HCC patients were ineligible for curative treatment including surgical resection or liver transplantation [10]. Moreover, the recurrence rate and distal metastasis are very high, and till now there is no effective therapeutic regimen for HCC patients [11]. Therefore, searching for a new strategy for HCC treatment in addition to surgery and chemotherapy becomes an essential matter for improving the survival rates and outcomes of HCC patients.

Curcumin is a polyphenol extracted from the root and rhizome of *Curcuma Longa*. It has various pharmacological properties including antioxidant [12], anti-inflammatory [13], and immune regulatory functions [14]. It also exhibits anticancer activity against many types of cancers including colon, stomach, lung, breast, and liver cancer [15–19]. The anticancer activity of curcumin is produced through regulating many signaling pathways and molecular targets which are essential for cancer development and progression, such as Wnt/β-catenin, PI3K/Akt, insulin-like growth factor (IGF), vascular endothelial growth factor (VEGF), as well as TGF-β1/smad3 [20–22]. Accordingly, curcumin was reported as a potential anticancer drug for HCC patients [11, 23–25]. However, still the exact anticancer mechanism of curcumin in HCC is not well understood [26].

Taurine (*2aminoethanesulfonic acid*) is a nonessential amino acid, synthesized primarily in the liver, kidney, and to a lesser extent, in the brain [27]. Its synthesis relies mainly on cysteine/methionine metabolism [28]. Additionally, it is not metabolized in the body and not implicated in gluconeogenesis, therefore it is not considered a direct energy source [29]. Taurine has been involved in a variety of biological functions such as mediating calcium balance, cell membrane stabilization, osmoregulation, bile acid conjugation, immune modulation, cytoprotective effect, as well as antioxidant and anti-inflammatory function [30–32]. It can also be used in clinics for the treatment of diabetes mellitus, cataract, cardiovascular, and hepatobiliary diseases [33]. Recent studies showed that taurine has an important antitumorigenic effect against lung, breast, and liver cancers as it can induce apoptosis of cancer cells through upregulation of Bax and p53 gene expression while downregulating the antiapoptotic Bcl-2 proteins [34–36].

Piperine is a plant alkaloid present in the *Piper nigrum L.* and the *Piper longum L.*, which are commonly used spices worldwide. Piperine has many beneficial functions to the body including antioxidant, antihypertensive, antidiabetic, anti-asthmatic, analgesic, antipyretic, anti-diarrheal, anti-inflammatory, anxiolytic, antispasmodic, hepato-protective, antidepressant, immunomodulatory, antithyroid, and finally antimutagenic [37–39]. Additionally, it is utilized commercially for the treatment of various diseases as it is used in the synthesis of some pharmaceutical products including antiseptic, antibacterial, insecticidal, diuretic, and those used for digestion disorders [40]. Moreover, it exhibits potent anticancer activity against many tumors such as melanoma, breast, and HCC through induction of oxidative stress as well as, stimulation of apoptosis through increased expressions of p53 and Bax protein, while suppressing cyclin A and Bcl-xL expression [41–44].

Piperine had been added to the curcumin to increase its bioavailability by decreasing the rate of its metabolic breakdown through inhibiting UDP-glucuronyl transferase, UDP-glucose dehydrogenase, CYP3A4, cytochrome BS, NADPH cytochrome, and aryl hydrocarbon hydroxylase (AAH) [37, 45]. Moreover, it had been reported that combining piperine and curcumin leads to enhancing the anticancer effect of the curcumin [46].

In recent years, dietary phytochemicals have been widely used for their potential anticancer properties, especially against HCC [47]. We had previously introduced an anticancer model formed of a combination of curcumin, piperine, and taurine (CPT) for HCC patients, and it had been assessed for its efficacy both in cell cultures and experimental animal models [48, 49]. The data revealed that the combination of curcumin and taurine achieved a synergistic effect in reducing the malignant transformation in HCC-induced rat liver, as well as reducing the serum levels of interleukin-2 (IL-2), interferon-gamma (IFN- γ), alpha fetoprotein (AFP), and alpha-fucosidase (AFU) rather than using curcumin or taurine alone. Therefore, curcumin and taurine combination may serve as a prophylactic agent in high-risk group patients exposed to chemical hepatocarcinogens [49]. Moreover, twenty HCC patients had been recruited in a single-arm, open-label phase II trial at the National Cancer Institute (NCI), Cairo University. The data showed that the combined effect of the CPT produced a significant decrease in the serum levels of IL-10, and miR-21, with a non-significant up-regulation of the miR-141 expression, that resulted in increasing the overall survival (OS) rates of the patients [50].

Therefore, the current study aimed to assess the immunotherapeutic effect of the CPT combination that was prepared in one capsule in HCC patients. This could help for finding a potential immunotherapeutic agent for HCC, which could reduce the aggressiveness of the disease and improve the clinical outcome of the patients.

## Materials and methods

This was a prospective single-arm phase II clinical trial included twenty-six HCC patients who were admitted to the HCC unit of the National Hepatology and Tropical Medicine Research Institute (NHTMRI) during the period between February 2021 and June 2021. All patients were diagnosed and confirmed for HCC by laboratory and pathological assessment, abdominal ultrasonography, triphasic CT abdomen, and MRI.

### Inclusion and exclusion criteria

The included patients in the study were those who failed the standard therapeutic approaches, had unresectable locally advanced or metastatic HCC, and they were not amenable to percutaneous ablation or trans arterial therapy, in addition to having adequate organ function.

Patients were excluded from the study if they had HIV infection, other malignancy, chronic use of systemic steroids or immunosuppressive drugs, brain metastases, active bleeding, pregnancy, or lactation, in addition to impairment of gastrointestinal function. Patients previously treated with systemic chemotherapy were enrolled in the study after a washout period of at least 4 weeks.

### Recruitment

The recruited patients were those who fulfilled the inclusion criteria with age varied from 50 to 80 years old, either males or females. All the HCC patients were subjected to full history taking, full clinical examination, as well as laboratory assessment in the form of complete blood count (CBC) analysis, coagulation profile, kidney function tests (serum urea and creatinine), and liver function tests including serum glutamic oxaloacetic transaminase (GOT), serum glutamic pyruvic transaminase (GPT), alpha-fetoprotein (AFP), albumin, lactate dehydrogenase (LDH), total and direct Bilirubin.

### Ethical considerations

The manuscript proposal was approved by the ethical committee of the NHTMRI, Cairo, Egypt which was in accordance with the 2011 declaration of Helsinki (approval no. NHTMRI-IRB 2–21). All patients were informed about the study and the nature of the administered drugs, where they signed a written informed consent before enrolment in the study.

### Experimental design

Twenty-six HCC patients were administered CPT capsules daily for three successive cycles, each cycle was 1 month. The daily amount of the CPT was 5 g of curcumin, 50 mg of piperine, and 500 mg of taurine divided into three doses after meals. The raw materials of CPT were obtained from (XI’AN Rongsheng Biotechnology CO., LTD, China), and the CPT capsules were manufactured in Egypt (Marcyrle manufacture). Each capsule was composed of 500 mg of curcumin, 5 mg of piperine, and 50 mg of taurine, where the recruited patients administered three capsules after breakfast, four after lunch, and three after dinner.

The doses were adjusted according to the allowed human safety profile, where curcumin and taurine have been approved by the US Food and Drug Administration (FDA) as “Generally Recognized As Safe” (GRAS) [50–52].

The primary endpoint was to evaluate the role of the combined CPT effect on the immunological competence of the recruited HCC patients. As patients were assessed for some immunological markers including CD4, CD8, CD25, interleukins levels of IL-2, IL-6, IL-12, IFN- γ, and Vascular endothelial growth factor (VEGF), in addition to the expression levels of forkhead box P3 (*FOXP3*) mRNA and *miRNA 21* after each cycle of administration in relation to the baseline level.

### Serological assessment

The enzyme-linked immunosorbent assay (ELISA) was used to assess the serum cytokine levels of IL-2 (BioLegend’s ELISA MAX™ Deluxe Set Catalog Number: 431,816), IL-6 (TX 77,494, USA-Cloud-Clone Corp and Catalog Number: SEA079Hu), IL-12 (BioLegend’s ELISA MAX™ Deluxe Set Catalog Number: 431,704), IFN- γ (TX 77,494, USA-Cloud-Clone Corp and Catalog Number: SEA049Hu) and VEGF-α (TX 77,494, USA-Cloud-Clone Corp and Catalog Number: SEA143Hu). In addition to the assessment of human CD4 (Novus, Catalog Number: NBP2-75145), human CD8 (QuickDetect TM, Catalog Number: 4418 − 100), and human CD25 (Human sCD25 / IL-2R ELISA KIT Catalog Number: 950.500.048) according to the manufacturer’s instructions.

### Assessment of the expression levels of FOXP3 mRNA and micro-RNA-21 (miRNA-21)

Total RNA was extracted from the whole blood cells using Direct-zol RNA Miniprep Plus (Cat# R2072, ZYMO RESEARCH CORP. USA) as recommended by the manufacturer’s instructions. The purity and the concentration of the extracted RNA were assessed using spectrophotometer nano-drop (Beckman, USA). Retro-transcription (cDNA) was performed using SuperScript IV One-Step RT-PCR kit (Cat# 12,594,100, Thermo Fisher Scientific, Waltham, MA USA) according to the manufacturer’s instructions.

The RT-PCR analysis was performed in 25 µl final volume with thermal reaction conditions of polymerase activation at 95 °C for 2 min, followed by 40 cycles of denaturation at 95 °C for 10s, annealing at 55 °C for 10s and extension at 72 °C for 30s. The primer sequences were as follow: *FOXP3* (F: CCTCTGTATGGTTGGCACCT, R: CCTTGCTCCAATTCCTCTCC).

*GAPDH* (F: TGGATTTGGACGCATTGGTC, R: TTTGCACTGGTACGTGTTGAT). *FOXP3* mRNA expression was quantified using Taqman Universal PCR Master Mix (SuperScript^™^ IV One-Step RT-PCR System). While the primer sequences for *miRNA-21* were (F: TGCTCGTAGCTTATCAGACTGATG, R: CAGTGCAGGGTCCGAGGTAT), and that of the reference miRNA-U6 was (F: GCTTCGGCAGCACATATACTAAAAT, R: CGCTTCACGAATTTGCGTGTCAT). The fluorescence was acquired and detected by StepOne Real-Time PCR System (Applied Biosystems, Foster City, CA, USA). The relative expression of *FOXP3* and *miRNA-21* was performed using the comparative Ct method (2^−ΔΔCt^), in which data were expressed as the fold change of *FOXP3* and *miRNA-21* expression in the patients normalized to the expression levels of the endogenous control (*GAPDH* and *U6;* respectively) [53].

### Assessment of the plasma levels of Curcumin, Piperine and Taurine in HCC patients

Sample extraction was done by placing 250 µl of patient plasma into a glass tube. Then 750 µl of acetonitrile was added. Tubes were mixed by vortex for 1 min and centrifuged at 10,000×*g* at 4 °C for 10 min. The supernatant was transferred to the high-performance liquid chromatography (HPLC) autosampler vials and was injected into the Liquid chromatography–mass spectrometry (LC–MS) system. The LC-MS system consisted of Agilent 1200 HPLC system (Agilent Technologies, CA, USA) coupled to ABSCIEX Q TRAP 3200 mass spectrometers (ABSCIEX, Germany) equipped with an electrospray ionization (ESI) interface. Data acquisition was performed with analyst 4.0 software (ABSCIEX). The working conditions for the separation of Taurine, Curcumin, and Piperine were illustrated in Table 1.

**Table 1:** The working condition for determination of Taurine, Curcumin and Piperine using HPLC/MS/MS

	Taurine	Curcumin	Piperine
Ion transitions	*m*/*z* 123.8:79.9	*m*/*z* 369.2:177.0	*m*/*z* 286.18:201.0
Run time	5 min	3 min	5 min
Retention time	2.81 min	1.5 min	0.88 min
Injected volume	5 µl	10 µl	10 µl
	0.2% formic acid in both Methanol: water (2:98, v/v).	0.1% formic acid in both acetonitril: water (50:50, v/v).	0.1% formic acid in both acetonitril: water (60:40, v/v).
Flow rate	250 µl/ min	500 ul/min	500 ul/min
Analytical column	Atlantis T3 (3 μm, 150 × 3 mm) reversed-phase analytical column (Waters, Ireland)	Agilent Poroshell 120-C18 (50 mm × 3 mm × 2.7 μm, Agilent)	Agilent Poroshell 120-C18 (50 mm × 3 mm × 2.7 μm, Agilent)
Reference method	Tang et al. [54]	Mei Wang et al. [55]	Mei Wang et al. [55]

**Fig. 1:**
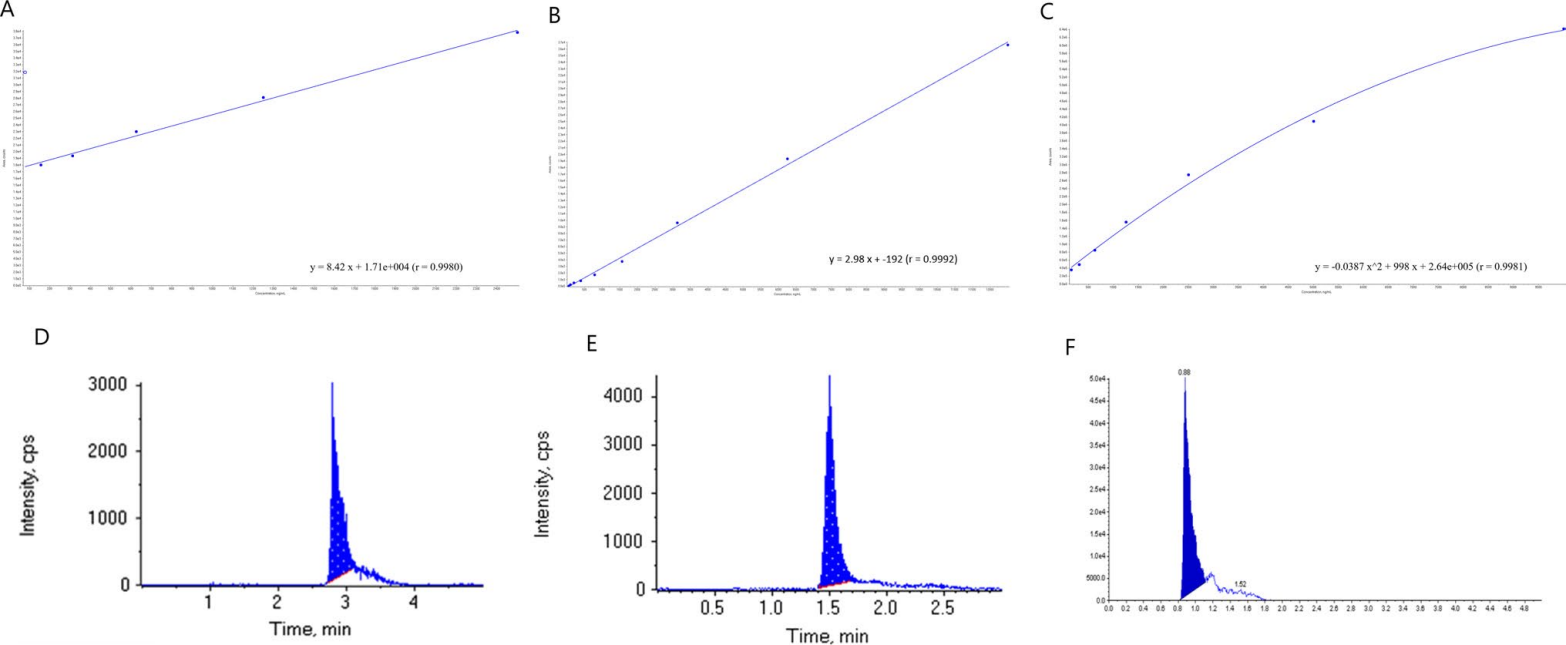
Calibration curve for **A** Taurine on concentration range (156–2500 ng/ml), **B** Curcumine on concentration range (203–13000 ng/ml), and **C** peperine on concentration range (157–10000 ng/ml). **D** Chromatograms for detection of Taurine at retention time 2.81 min, **E** Curcumine at retention time 1.5 min, and **F** Piperine at retention time 0.88 min

Serial dilutions of standards were prepared at concentrations ranged from 156 to 2500 ng/ml for Taurine, 203–13000 ng/ml for Curcumin, and 157–10000 ng/ml for piperine in drug-free media. They were extracted as mentioned in sample preparation to make a calibration curve [54, 55], (Fig. 1**).**

### Statistical analysis

Data were analysed using SPSS package (version 22; SPSS Inc., Chicago, IL, USA). categorical data were presented as frequencies (percentages), while numerical data were presented as mean ± SD or median and interquartile range (IQR) according to the normality of data distribution. Comparisons between variables were done using ANOVA for repeated measures followed by Bonferroni correction for adjustment of multiple comparisons or Friedman’s test followed by LSD post hoc for multiple comparisons as appropriate.

## Results

The current study included 26 HCC patients with a median age of 62 years (range: 50–76). Males represented 69.2% (18/26) and females were 30.8% (8/26). All patients were positive for HCV, and 42.3% (11/26) of them showed distant metastasis. There were 69.2% (18/26) patients with CHILD-A, and 30.8% (8/26) were CHILD-B. patients were classified according to Barcelona Clinic Liver Cancer (BCLC) classification into stage A in 5 (19.2%) patients, stage B in 8 (30.8%) patients, stage C in 8 (30.8%) patients, and stage D in 5 (19.2%) patients. At the end of the study, 9 (34.6%) patients died and 17 (65.4%) were alive. The other clinical features of the patients were summarized in Table 2.

**Table 2:** Clinical features of the assessed HCC patients

Assessed parameters		HCC patients (n = 26)	Assessed parameters		HCC patients (n = 26)
Age (years)	mean ± SD	62 ± 6.1	Sex	Male	18 (69.2%)
	Median (IQR)	62 (50–76)		Female	8 (30.8%)
Smoking	No	9 (37.5%)	DM	No	15 (57.7%)
	Yes	15 (62.5%)		Yes	11 (42.3%)
HTN	No	20 (76.9%)	Cardiac disease	No	26 (100%)
	Yes	6 (23.1%)		Yes	0 (0.0%)
HCV	yes	26 (100%)	Metastasis	no	15 (57.7%)
	No	0 (0.0%)		yes	11 (42.3%)
number of nodules	1	15 (57.7%)	BCLC	A	5 (19.2%)
	2	4 (15.4%)		B	8 (30.8%)
	3	2 (7.7%)		C	8 (30.8%)
	multiple	5 (19.2%)		D	5 (19.2%)
Child	A	18 (69.2%)	Lap ablation	No	22 (84.6%)
	B	8 (29.8%)		Yes	4 (15.4%)
Nexavar	No	26 (100%)	TACE	No	17 (65.4%)
	Yes	0 (0.0%)		Yes	9 (34.6%)
Death	alive	17 (65.4%)	Sup-med	No	13 (50.0%)
	died	9 (34.6%)		Yes	13 (50.0%)

*BCLC* Barcelona clinic liver cancer, *DM* diabetes mellitus, *HCV* Hepatitis C virus, *HTN* Hypertension

### Effect of CPT on the immunological profile of the patients

**Fig. 2:**
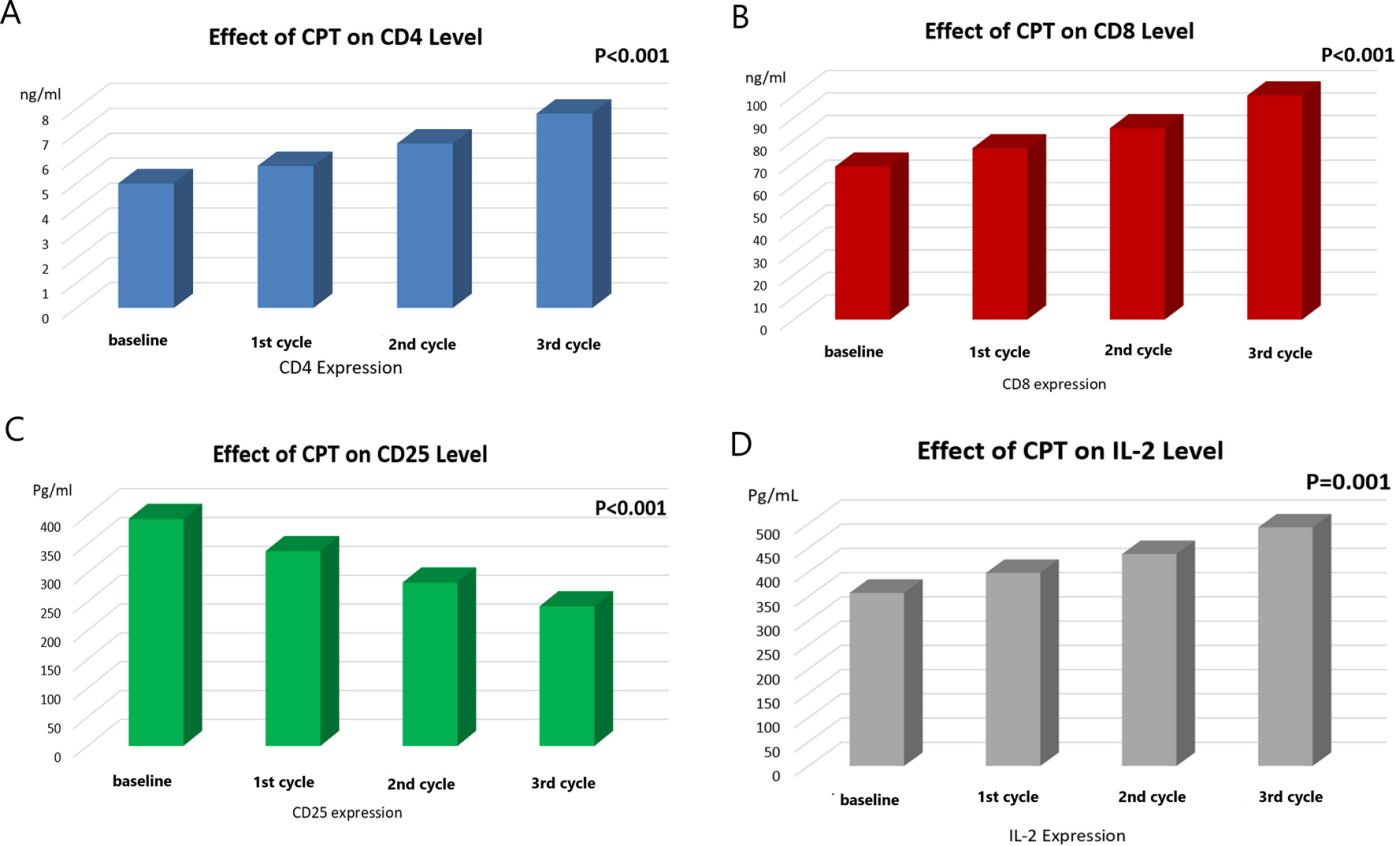
The effect of curcumin, piperine and taurine (CPT) on the level of **A** CD4, **B** CD8, **C** CD25 and **D** Interleukin-2 in HCC patients

As shown in Table 3, there was a significant increase in the plasma levels of CD4 and CD8 after the second and third cycle of the CPT, however there was no significant change after the first cycle. The plasma level of CD4 at baseline and after the first, second and third cycle were [5 ± 1.2 ng/ml, 5.7 ± 1.2 ng/ml, 6.6 ± 1.4 ng/ml, and 7.8 ± 1.8 ng/ml; respectively, P < 0.001, Fig. 2A]. While the plasma level of CD8 at baseline and after the first, second and third cycle were [68.2 ± 10.9 ng/ml, 76.4 ± 9.4 ng/ml, 85.3 ± 12.6 ng/ml, and 99.9 ± 20.6 ng/ml; respectively, P < 0.001, Fig. 2B]. On the other hand, there was a significant decrease in the plasma level of CD25 after the first, second and third cycle [337.7 ± 50 pg/ml, 283.2 ± 39 pg/ml, and 242 ± 51 pg/ml; respectively] compared to the baseline level [393.5 ± 66 pg/ml, P < 0.001, Fig. 2C].

**Table 3:** The impact of CPT on the immunological and biological parameters of the HCC patients

	Baseline	1st cycle	2nd cycle	3rd cycle	P value
Immunological parameters					
CD4 (ng/ml)	5 ± 1.2^a^	5.7 ± 1.2^a^	6.6 ± 1.4^b^	7.8 ± 1.8^c^	P < 0.001
CD8 (ng/ml)	68.2 ± 10.9^a^	76.4 ± 9.4^a^	85.3 ± 12.6^b^	99.9 ± 20.6^c^	P < 0.001
CD25 (pg/ml)	393.5 ± 66^a^	337.7 ± 50^b^	283.2 ± 39^c^	242 ± 51^d^	P < 0.001
IL2 (pg/ml)	357 ± 156^a^	398 ± 140^b^	437 ± 147^b^	492 ± 156^c^	P = 0.001
IL12 (pg/ml)	462 ± 85^a^	519 ± 108^ab^	563 ± 135^b^	609 ± 175^b^	P = 0.006
IL6 (pg/ml)	345 ± 26^a^	299 ± 30^b^	247 ± 48^c^	214 ± 57^d^	P < 0.001
IFN-γ (pg/ml)	367 ± 28^a^	377 ± 51^a^	402 ± 92^b^	437 ± 145^c^	P = 0.029
FOXP3	5.3 ± 0.8	4.2 ± 0.76	3.2 ± 0.67	2.5 ± 0.79	P = 0.184
miRNA21	5.5 ± 0.88^a^	4.1 ± 0.78^b^	3 ± 0.75^c^	2.5 ± 0.76^d^	P < 0.001
VEGF (pg/ml)	407 ± 55^a^	342 ± 80^b^	285 ± 82^c^	246 ± 92^d^	P < 0.001
Liver function tests					
AFP (ng/dl)	37 (1–4298)^a^	60 (2–3000)^a^	56 (2–2500)^b^	52 (2–2300)^c^	0.004
LDH (µl/l)	341 (223–892)^a^	357 (245–699)^a^	287 (204–501)^b^	283 (194–550)^b^	0.020
Albumin (g/dl)	3.6 ± 0.72	3.7 ± 0.66	3.6 ± 0.54	3.6 ± 0.63	0.738
GPT (µl/l)	45 (14–185)	39 (17–126)	39 (14–141)	44 (20–136)	0.947
GOT (µl/l)	56 (20–136)	48 (18–238)	52 (13–170)	53 (26–189)	0.933
Total bilirubin (mg/dl)	1.3 (1–11)	1 (0–3)	1.2 (1–3)	1 (1–2)	0.206
Direct bilirubin (mg/dl)	0.45 (0–6)^a^	0.25 (0–2)^ab^	0.35 (0–2)^ab^	0.33 (0–1)^b^	0.011
Hematological parameters					
Hb (g/dl)	12.4 ± 2.18	12.3 ± 2.15	12.3 ± 2.26	12.3 ± 2.59	0.883
RBCs (x10^9^/L)	4.4 ± 0.63	4.2 ± 0.83	4.3 ± 0.66	4.3 ± 0.72	0.184
WBCs (x10^9^/L)	5.96 ± 2.1	5.97 ± 3.1	5.44 ± 2.8	5.90 ± 2.8	0.572
Platelets (x10^9^/L)	142 (63–378)	143 (80–444)	136 (92–431)	151 (72–377)	0.870
PT	13.5 ± 0.67	13.5 ± 1.01	13.6 ± 0.96	13.4 ± 0.63	0.282
INR	1.1 ± 0.08	1.1 ± 0.09	1.1 ± 0.12	1 ± 0.07	0.408
Kidney function tests					
Creatinine (mg/dl)	0.9 ± 0.2	1 ± 0.2	1.1 ± 0.3	1 ± 0.3	0.192
Urea (mg/dl)	28.1 ± 9.7	30.8 ± 9.2	31.2 ± 9.3	28.7 ± 11.5	0.695
CPT plasma levels					
Taurine (ng/ml)	59 (20–1520)^a^	125 (26–2170)^b^	231 (30–5890)^bc^	708 (20–5380)^c^	P = 0.036
Curcumin (ng/ml)	0.83 ± 0.42^a^	67.2 ± 5.9^b^	71.4 ± 7.1^b^	72.1 ± 6.5^b^	P = 0.037
Piperine (ng/ml)	49 (0.2–1160)^a^	688 (0–2510)^b^	1040 (0.2–2360)^b^	662 (0.1–3650)^b^	P < 0.001

*AFP* Alpha fetoprotein, *CD* cluster of differentiation, *CPT* curcumin, piperine and taurine, *GOT* glutamic oxaloacetic transaminase, *GPT* Glutamic pyruvic transaminase, *Hb* Haemoglobin concentration, *IFN-γ* Interferon gamma, *IL-2* interleukin-2, *INR* international normalized ratio, *LDH* Lactate dehydrogenase, *PT* prothrombin time, *RBCs* Red blood cells, *VEGF* Vascular endothelial growth factor, *WBCs* White blood cells. P < 0.05 is statistically significant. Variables with different letters are significantly different

**Fig. 3:**
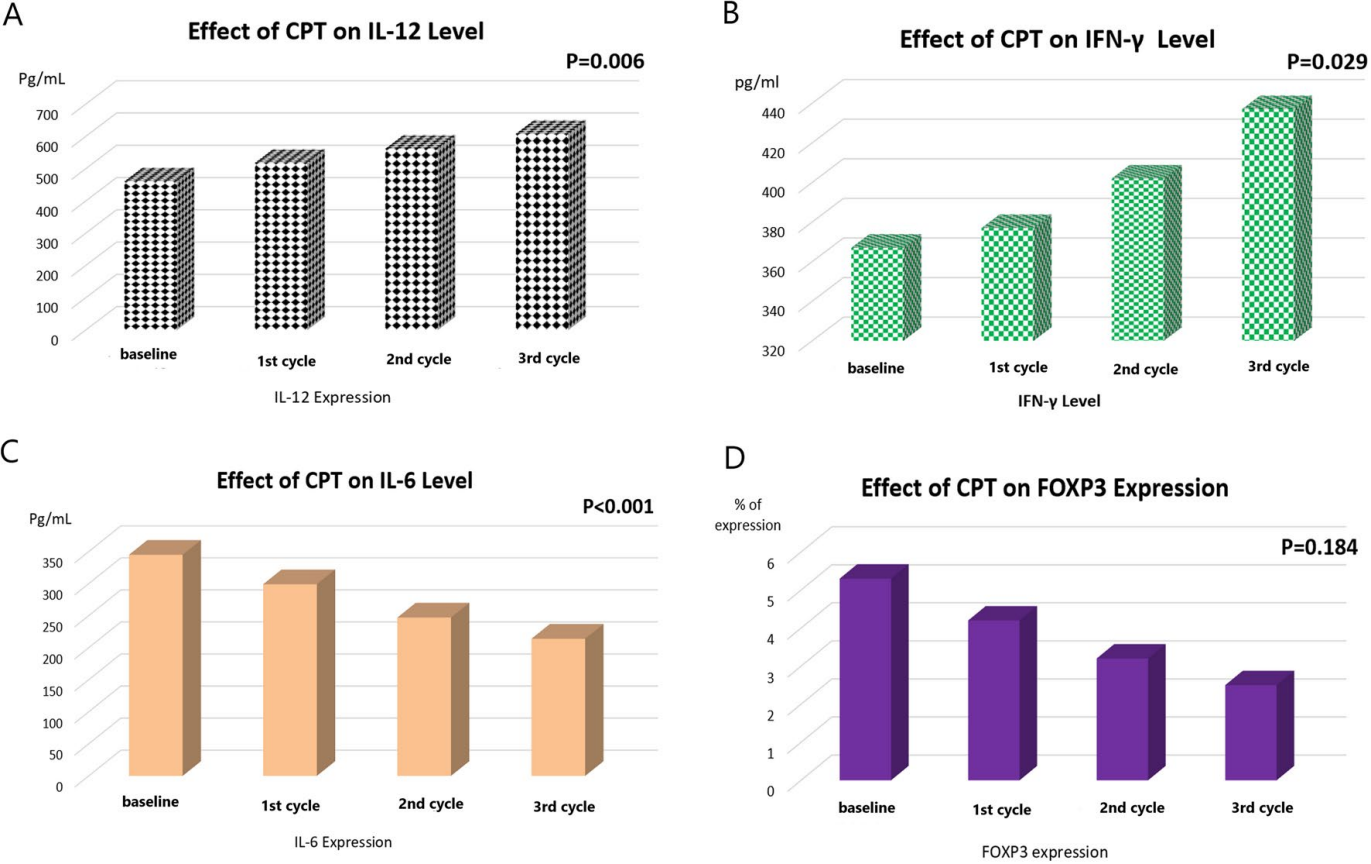
The effect of curcumin, piperine and taurine (CPT) on the interleukin level of **A** IL-12, **B** Interferon gamma (IFN- γ) and **C** IL-6, and **D** *FOXP3* mRNA expression levels in HCC patients

Regarding the cytokine levels of the assessed HCC patients, there was a significant increase in the serum level of IL-2 after the first, second and third cycle of the tested CPT [398 ± 140 pg/ml, 437 ± 147 pg/ml, and 492 ± 156 pg/ml; respectively], in comparison to the baseline level [357 ± 156 pg/ml, P = 0.001]. However, there was no significant change between the first and the second cycle (Fig. 2D). There was a significant increase in the serum level of IL-12 after the second and third cycle of the tested CPT compared to the baseline level, however there was no significant change in the IL-12 level between the three cycles [baseline: 462 ± 85 pg/ml, 1st cycle: 519 ± 108 pg/ml, 2nd cycle: 563 ± 135 pg/ml, and 3rd cycle: 609 ± 175 pg/ml, P = 0.006, Fig. 3A]. Also, there was a significant increase in the serum level of IFN- γ after the second and third cycles of the CPT, however there was no significant change after the first cycle, compared to the baseline level [baseline: 367 ± 28 pg/ml, 1st cycle: 377 ± 51 pg/ml, 2nd cycle: 402 ± 92 pg/ml, and 3rd cycle: 437 ± 145 pg/ml, P = 0.029, Fig. 3B]. On the other hand, there was a significant decrease in the serum level of IL-6 after the first, second and third cycle of the CPT [299 ± 30 pg/ml, 247 ± 48 pg/ml, and 214 ± 57 pg/ml; respectively, P < 0.001], in comparison to the baseline level [345 ± 26 pg/ml, Fig. 3C].

### The impact of the CPT on the expression levels of *FOXP3* mRNA and *miRNA-21*

**Fig. 4:**
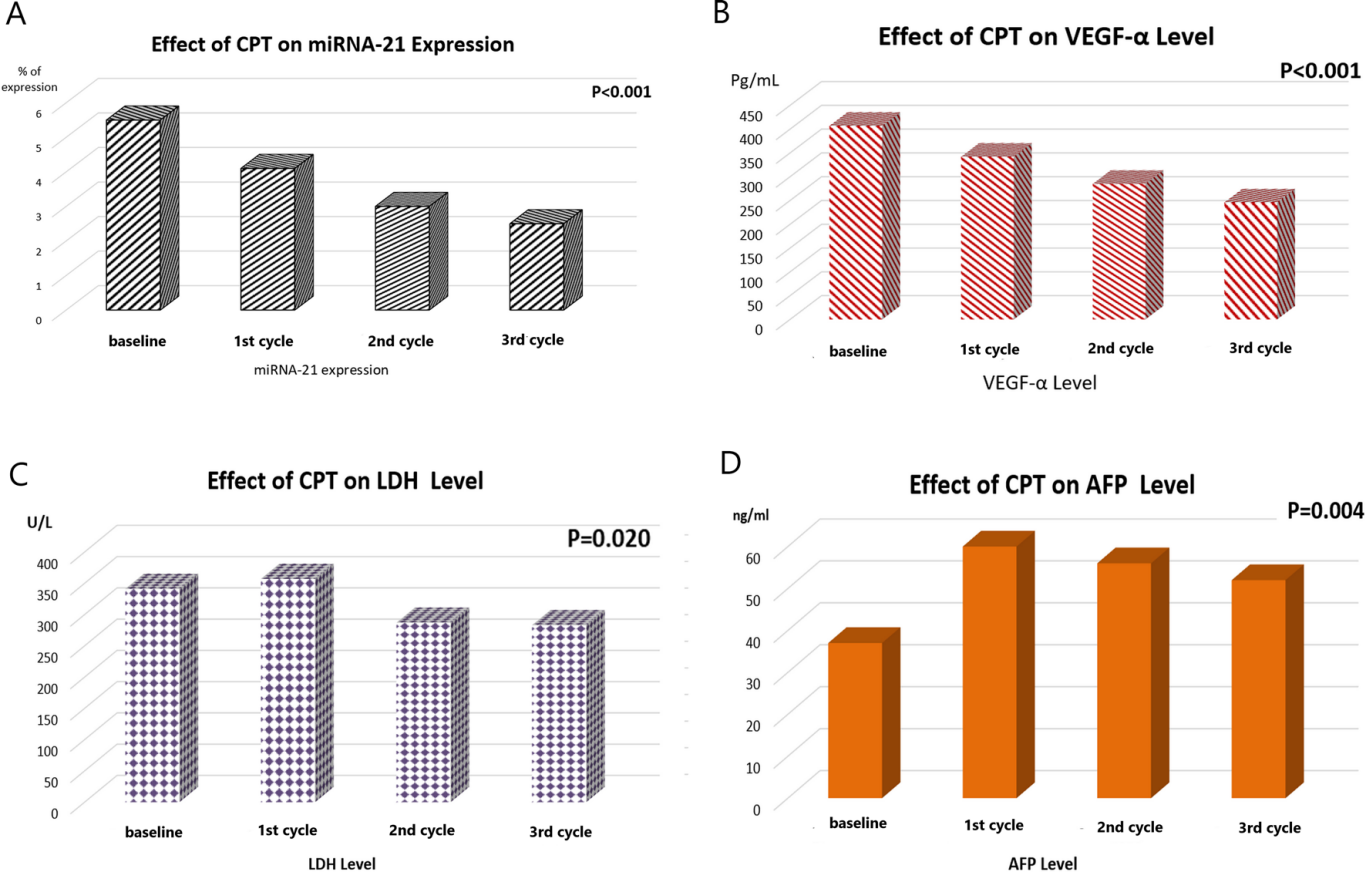
The effect of curcumin, piperine and taurine (CPT) on the expression levels of **A** *miRNA-21*, **B** Vascular endothelial growth factor (VEGF), **C** lactate dehydrogenase (LDH) and **D** alpha-fetoprotein (AFP) in HCC patients

There was no significant change in the *FOXP3* gene expression before and after CPT administration, though there was a noticeable decrease in its level after each cycle, however it did not reach a significant level [baseline: 5.3 ± 0.8, 1st cycle: 4.2 ± 0.76, 2nd cycle: 3.2 ± 0.67, and 3rd cycle: 2.5 ± 0.79; respectively, P = 0.184, Fig. 3D]. Meanwhile, the expression level of *miRNA-21* was significantly decreased after the first, second and third cycle [4.1 ± 0.78, 3 ± 0.75, and 2.5 ± 0.76; respectively, P < 0.001], in comparison to the baseline expression level [5.5 ± 0.88, Fig. 4A].

### Effect of CPT on the serum levels of VEGF-α and LDH

There was a significant decrease in the serum level of VEGF-α after the first, second and third cycle of the CPT [342 ± 80 pg/ml, 285 ± 82 pg/ml, and 246 ± 92 pg/ml; respectively, P < 0.001], in comparison to the baseline level [407 ± 55 pg/ml, Fig. 4B]. The serum level of LDH was also decreased after the second and third cycle of CPT in comparison to the baseline level [baseline: 341(223–892) µl/l, 1st cycle: 357(245–699) µl/l, 2ns cycle: 287(204–501) µl/l, and 3rd cycle: 283(194–550) µl /l, P = 0.020, Fig. 4C].

### Effect of CPT on the liver function tests

The AFP was significantly decreased after the second and third cycle of CPT compared to the baseline level [baseline: 37(1-4298), 1st cycle: 60(2-3000), 2ns cycle: 56(2-2500), and 3rd cycle: 52(2-2300) ng/dl, P = 0.004, Fig. 4D]. Similarly, the serum level of direct bilirubin was significantly decreased after the third cycle of CPT, however there was no significant change after the first and the second cycle [baseline: 0.45 (0–6), 1st cycle: 0.25 (0–2), 2ns cycle: 0.35 (0–2), and 3rd cycle: 0.33 (0–1) mg/dl, P = 0.011]. On the other hand, there was no significant impact of CPT on GOT, GPT, total bilirubin, albumin, PT and INR. The data were shown in Table 2.

### Plasma levels of curcumin, piperine and taurine in HCC patients

**Fig. 5:**
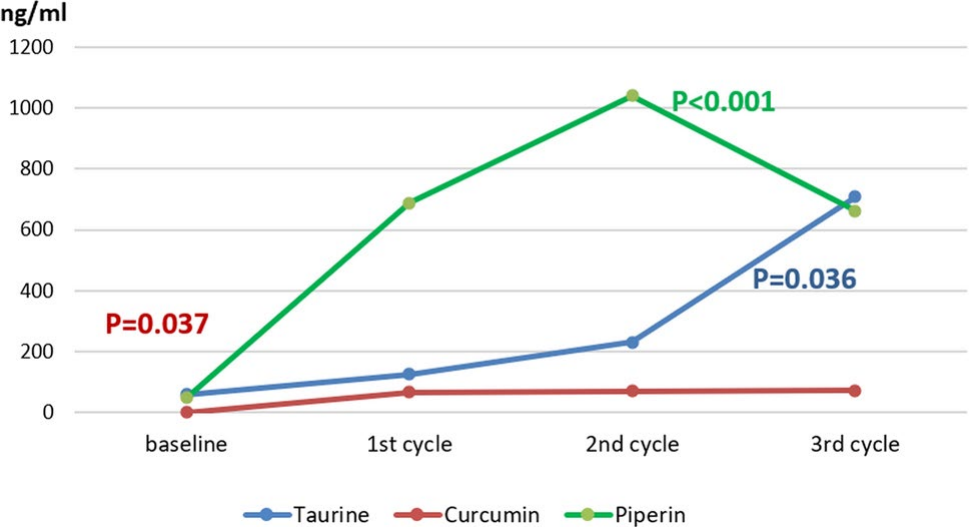
Plasma levels of curcumin, piperine and taurine in HCC patients after three cycles of administration

The plasma levels of taurine, piperine and curcumin were significantly elevated after administration of the CPT combination in relation to the baseline levels (P = 0.036, P < 0.001, and P = 0.037; respectively). However, there was no significant change in the plasma levels of piperine and curcumin among the three doses Fig. 5.

## Discussion

The current study was performed to assess the immune-potentiating effect of CPT on 26 HCC patients who failed other lines of HCC treatment. Each component of the proposed CPT therapy had been recognized for its potent anticancer activity against HCC cells [23, 36, 44]. However, their combination together has not been assessed in HCC patients yet. To the best of our knowledge, it is the first time to prepare a combination of curcumin, piperine, and taurine in one capsule. So that it would be more effective for the patients.

The present data revealed that patients administered CPT for 3 months showed increased plasma levels of CD4 and CD8 after the second and the third dose of the CPT, with a significant increase in the serum levels of IL-2, IL-12, and IFN-γ. It has been shown that the peripheral blood numbers of CD4+, CD8 + T lymphocytes, and T-regs are important indicators for the activity of the immune system against cancer [56]. Therefore, higher numbers of CD4 + and CD8 + T cells indicate more lymphocyte infiltration into the tumor tissue, which lead to tumor regression. While an increased number of T-regs is associated with suppression of the anti-tumor immune response and consequently tumor cell proliferation [57, 58].

These data are supported by Bhattacharyya et al., and Fu et al., who reported that curcumin can stimulate proliferation and activation of CD4 + and CD8 + cells in the tumor microenvironment (TME) and switching Th2 cytokine towards Th1-type response [59, 60]. Also, Churchill et al. [61] reported that curcumin stimulates Th1-type immune responses and upregulates IFN-g mRNA expression.

Moreover, Guo and his team developed a natural Chinese curcumin-based medicine for HCC patients in which curcumin was delivered to the tumor site through loading with glycyrrhetic acid (GA)-APS-disulfide bond-DTA-Cur nanomicelle (GACS-Cur). They observed that GA-APS-DTA-Cur nanomicelle had anticancer properties in addition to the immunostimulatory action in the form of increased IL-2, TNF-α and IFN-γ expression as well as increased CD4 + T and CD8 + T cell infiltration [62].

Additionally, the present data showed that patients administered CPT had a significant decrease in the plasma level of CD25 and Il-6 after the first, second and third cycle of treatment compared to the baseline level. Meanwhile, there was no significant change in the *FOXP3* gene expression before and after CPT administration. Though there was an observed decrease in *FOXP3* expression level after each cycle, however it did not reach a significant level. These data are in agreement with Zhao et al., who found that curcumin can effectively increase the anticancer immune response through reducing Treg cell population, IL-10 and TGF-b levels. While in comparable to the present results, they observed that curcumin reduces both the protein and mRNA levels of *CTLA4* and *FOXP3* [63].

Furthermore, the expression level of miRNA-21 was significantly decreased after the first, second and third cycle of CPT treatment in comparison to the baseline expression level. The *miRNA-21* was demonstrated to promote the proliferation, migration, and invasion of liver cancer cells through inhibiting FASLG, SOCS6, and KLF5 [64–66]. In consistent with these results, Wang et al. found that higher *miRNA-21* expression associated significantly with shorter post-operative survival rates in patients with HCC [67]. Moreover, Li et al. [26], reported that curcumin has anticancer activity through downregulating *miRNA-21* which leads to inhibition of tissue inhibitor of metalloproteinase 3 (*TIMP3)* through targeting TGF-β1/smad3 signalling pathway [26].

The present study also demonstrated that there was a significant decrease in the serum levels of VEGF-α after the first, second, and third cycle of the CPT in comparison to the baseline level. The VEGF-α is the main regulatory factor for neovascularization (angiogenesis) which is an essential mechanism for tumor proliferation and metastasis. In line with these data, Guo et al. [62], found that in vivo and in vitro assessment of GA-APS-DTA-Cur nanomicelle revealed downregulation of VEGF-α which indicated that curcumin has an anti-metastatic activity.

Moreover, the current work showed that CPT administration could efficiently improve the hepatic functions and downregulate HCC markers indicated by the significant decrease of the serum levels of AFP after the second and third cycles of CPT compared to the baseline level. Similarly, the serum level of direct bilirubin was significantly decreased after the third cycle of CPT, in addition to the significant decrease in the serum level of LDH. These data are concomitant with that of Faloppi et al. [68], who reported that curcumin could protect against HCC through reducing the AFP level in the tumor tissue of the experimental animal model. Also, they reported that curcumin could inhibit the anaerobic glycolysis by decreasing the LDH and hypoxia-inducible factor 1 (HIF-1) in the tumor tissue. It had been proven in a preclinical model that hypoxia stimulates the production of HIF-1 which accordingly increased LDH and VEGF production through stimulating the anaerobic glycolytic metabolism, and angiogenesis in the cancer microenvironment [69, 70].

It is well known that curcumin had a low pharmacokinetic profile due to its poor bioavailability in humans, even when administered at high doses [71]. Many previously published studies reported the beneficial effect of combining piperine and curcumin together [72, 73]. Anand et al., demonstrated that the association of 2 g of curcumin and 5 mg of piperine showed a 3-fold increase in the pharmacological properties of curcumin [74]. Therefore, we thought to add piperine and taurine to improve the bioavailability of the curcumin and allow for more synergistic anticancer, antioxidant, and immunomodulatory effects of the three compounds together.

In conclusion, the current study provided evidence that CPT treatment could potentially enhance the anti-tumour immune response in HCC patients through increasing the plasma levels of CD4, CD8, IL-2, IL-12, and IFN-γ after CPT administration. While there was a significant decrease in the plasma levels of CD25, IL-6, VEGF-α, LDH, AFP, and *miRNA-21* expression compared to baseline levels. Therefore, further studies are highly recommended to evaluate the immunomodulatory action of CPT in depth on a larger number of HCC patients with different grades and stages. Also, it is highly required to assess the effect of CPT on different types of cancer. This will pave the way to add other modalities for managing cancer patients using natural compounds with minimal side effects in relation to the other anticancer drugs.

### Supplementary Information

Supplementary material 1

Supplementary material 2

## Data Availability

All data generated or analysed during this study are included in this published article.
