# Supplementary figures and images for: The potential immuno-stimulating effect of curcumin, piperine, and taurine combination in hepatocellular carcinoma; a pilot study
Source: Discov Oncol. 2023 Sep 13;14:169. doi: 10.1007/s12672-023-00785-1 (PMC10499730; doi:10.1007/s12672-023-00785-1)

## The Consort Flowchart

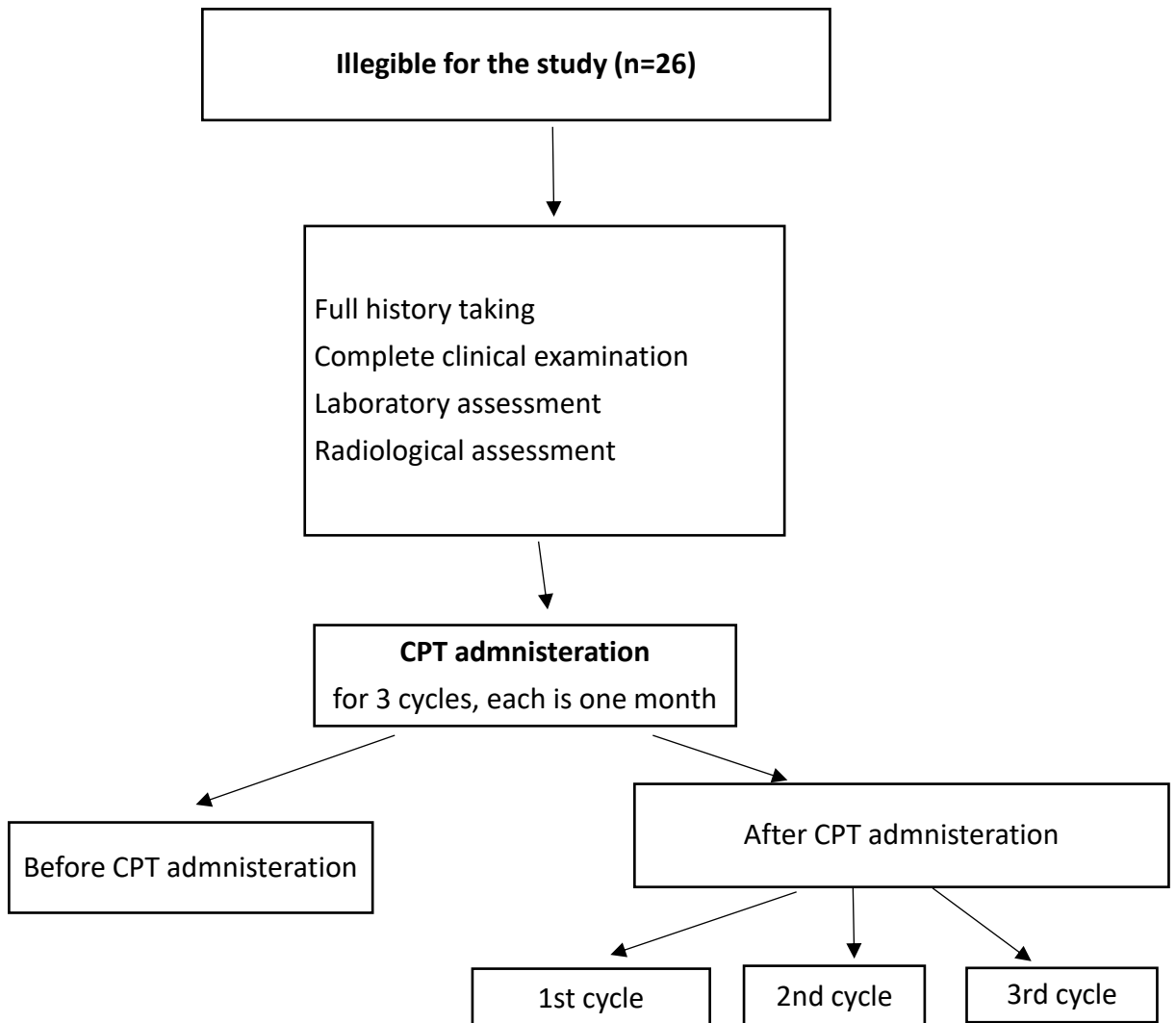

Supplement: Supplementary file 1 — Supplementary material 1 [file 12672_2023_785_MOESM1_ESM.pdf]
