# Supplementary material for: The potential immuno-stimulating effect of curcumin, piperine, and taurine combination in hepatocellular carcinoma; a pilot study
Source: Discov Oncol. 2023 Sep 13;14:169. doi: 10.1007/s12672-023-00785-1 (PMC10499730; doi:10.1007/s12672-023-00785-1)
Supplement: Supplementary file 2 — Supplementary material 2 [file 12672_2023_785_MOESM2_ESM.docx]

**Role of Curcumin, Piperine and Taurine adminstration in the Treatment of Patients with Hepatocellular Carinoma**

**Protocol for a Clinical Trial**

**Clinical PI**

**-**

**Medical oncology Dept.**

**National Cancer Institute**

**Cairo University**

**Laboratory PI**

**Dr. Motawa E. El-Houseini**

***Prof.Medical Biochemistry***

***and molecular Biology***

***Cancer Biology Dept.***

***National Cancer Institute,***

***Cairo University***

**رول الكوركومين و البيبرين و التورين في علاج سرطان**

**الكبد**

**بروتوكول دراسة اكلينيكية**

**الباحث الرئيسي الاكلينيكي**

**المعهد القومي للأورام، جامعة القاهرة**

**الباحث الرئيسي المعملي**

**أ.د. مطاوع الحسيني**

**أستاذ بيولوجيا الاورام**

**المعهد القومي للاورام، جامعة القاهرة**

**Table of contents**

| **Section** | **Page** |
| --- | --- |
|  |  |
| General Information | 4 |
|  |  |
| Abstract/synopsis | 5 |
|  |  |
| Introduction | 6 - 8 |
|  |  |
| Aim | 9 |
|  |  |
| Methods | 10 |
|  |  |
| References | 11-14 |
|  |  |
|  |  |

**General Information:**

- ***The principle investigators names:***
- ***Prof. Dr. Motawa E. EL-Houseini***
- ***Dr.***
- -Address: Dept. Cancer Biology and Medical oncology NCI, Cairo University
- ***The sponsor/Source of grant:*** The Egyptian National Cancer Institute,
- Cairo University, Egypt
- Address: Fom El Khalig Square, Cairo , Egypt
- Telephones: +20225328286
- website: <http://www.nci.cu.edu.eg/>
- **The trial sites and** clinical laboratories
- Address: The Egyptian National Cancer Institute, Fom El Khalig Square, Cairo, Egypt
- Telephones: +20225328286
- website: <http://www.nci.cu.edu.eg/>

**Abstract/synopsis**

**Introduction*:*** Worldwide, hepatocellular carcinoma (HCC) is a common cancer secondary to hepatitis viruses (HBV and HCV), alcohol uptake and food contamination with aflatoxins and the resultant cirrhosis. HCC is the most common cancer among Egyptian males secondary to HCV epidemic. Generally, HCC carries a very poor prognosis. Currently, only the drug “Sorafenib” is approved for the treatment of advanced HCC that is not amenable to surgery and other local or regional therapies. This drug delays tumor progression for few months at the expense of noticeable side effects and a very high cost that prevents its widespread use particularly in low-socioeconomic countries like Egypt. “**Curcumin** (C)” is a natural product of Tumeric roots that is used as food additive for centuries. Preclinical and clinical studies have shown its efficacy against many tumor types. It proved to be reasonably tolerated and at a low cost. “**Taurine** (T)” is a food supplement that increases efficacy of curcumin when added to it. “**Piperine** (P)” is a food additive that improved the sparse absorption of curcumin.

**Aims:** To evaluate the safety and efficacy of Curcumin, Piprine and Taurine in advanced HCC patients

**Methods:** This is a prospective, two-stage two-arm single-center phase II clinical trial. It will include patients with advanced HCC who will receive a combination of Curcumin (C) , Piprine (P) and Taurine (T). Initially 50 patients will be included per arm. The study is expected to recruit the initial 50 patients in 3 months (stage I). If the study to be extended (stage II), another 3 months will be needed. An additional 6 months for follow up will be allowed. The overall duration may be 12 months.

**Expected results:** It is expected that the previous mentioned combination will produce results comparable to approved drugs in terms of disease stabilization and progression free and overall survival.

**The outcome and conclusion:** if this study proves as usefull treatment, this will provide an alternative treatment for an advanced HCC with good safety profile. It will carry huge cost saving for the NCI and Egypt. It may be widely used on the international level.

**Key words**: HCC patients, Curcumin, Piperine and Taurine treatment, biomarkers

**I. Introduction**

**Background**

Worldwide, hepatocellular carcinoma (HCC) is the fifth most common cancer and the third most common cause of cancer-related deaths. HCC is considered to be a major health problem in Asia and Africa. In Egypt, HCC accounts for about 4.7% of chronic liver disease patients. HBV and HCV infections are strongly associated with liver cirrhosis and HCC [1]. Conventional therapies of the early stages include surgical resection, liver transplantation, radiofrequency ablation, and ethanol injection with a 5-year overall survival rates of 50–70%. Unfortunately, most patients (70–85 %) present with an advanced or unrespectable disease with limited therapeutic options [2,3].

Curcumin (diferuloylmethane) is the chief component of the spice turmeric and is derived from the rhizome of the East Indian plant *Curcuma longa* [4]. It is a very potent antioxidant with hepatoprotective activity [5,6]. For instance in rat models, curcumin pretreatment produced protective effects of carbon tetra-chloride (CCL4) induced acute liver failure [7]. In numerous other studies, curcumin has been shown to scavenge oxygen free radicals; it prevents lipid peroxidation and increase intracellular glutathione concentrations in rat hepatocytes [8, 9, 10,11]*.* Curcumin as a polyphenol compound demonstrates anti-inflammatory and antioxidant activities. It also exhibits anti-survival, anti- proliferative, anti-invasive and antiangiogenic activities against cancer cells [12, 13]. These effects are mediated in part through the down regulation of various transcription factors including nuclear factor NF-KB [14, 15].

Animal studies have revealed that curcumin can prevent carcinogen-induced tumorigenesis and inhibit growth of implanted human tumors [16]. Such studies have led to clinical trials of curcumin in patients with colon cancer familial adenomatous polyposis (Fap), pancreatic cancer and multiple myeloma. The non-toxic nature of curcumin, as well as its multiple beneficial clinical effects, has made it one of the most attractive compounds to be explored for chemoprevention of cancers [17, 18, 19, 20,].

However, poor water solubility and limited oral bio-availability of curcumin are *the major road blocks in its development as a therapeutic drug for cancer and other* chronic diseases . Piperine is a pungent alkaloid present in Piper nigrum Linn, and P. longum Linn. Piperine is absorbed very fast across the intestinal barrier. It may act as an apolar molecule and form apolar complex with drugs and solutes. It may modulate membrane dynamics due to its easy partitioning thus helping in efficient permeability across the barriers [,21,)*.* It increases the bioavailability of curcumin. A 20 mg dose of piperine can increase the bioavailability of curcumin by 20-fold in humans [22].

Curcumin has a protective effect against chemically induced experimental hepatocarcinogenesis [ 23,24]. It also prevents acute liver damage by at least two mechanisms: acting as an antioxidant and by inhibiting NF-kB activation and the subsequent production of pro-inflammatory cytokines [25]. Curcumin attenuates lipo-polysaccharide induced hepatotoxicity by preventing cytotoxic effects of oxygen free radicals and cytokines [26, 27, 28].

The inhibition of tumor formation by curcumin has been attributed to its anti-initiation and anti-promotion [29,30]. However, a prepared biodegradable nono-particle formulation of curcumin can suppress cancer cell proliferation, induce apoptosis, inhibit angiogenesis, and suppress the expression of anti-apoptotic proteins [31].

Curcumin proved to be a potent immunomodulatory agent that can modulate the activation of T cells, B cells, macrophages, neutrophils, natural killer cells and dendritic cells. Curcumin can alsodown regulate the expression of various pro-inflammatory cytokines presented by TNF, interleukin -1 (IL-1), IL-2, IL-6, IL-8 and IL-12 through inactivation of transcription factor NF-KB [32]. Curcumin is emerging as a potential therapeutic compound in chronic liver diseases, being a major cause of morbidity and mortality worldwide. It exerts beneficial effects in animal models of liver injury and cirrhosis that are considered major risk factors for the development of HCC [33]. Curcumin is generally recognized by the FDA as a safe food additive up to 8 gm/day [34].

Taurine is one of the end-products of sulphur metabolism. In the human body, taurine is synthesized from the essential amino acid methionine and its related amino acid cysteine [35]. Its synthesis requires pyridoxal-phosphate that is the active coenzyme form of vitamin B6. Additionally, vitamin B6 deficiency has been shown to impair taurine synthesis [36].. Taurolidine (a taurine derivative) was shown to possess significant antineoplastic and antiproliferative activities in several human tumor cell lines *in vitro.* Taurolidine also decreased the tumor burden in a murine model of intra-peritoneal (I.P.) human ovarian carcinoma cells. Furthermore, anti-neoplastic evaluation of taurolidine in nude mice bearing i.p. xenografts of human ovarian tumor cells demonstrated that this agent significantly inhibits tumor development and growth [37 ]. In HCC patients, taurine ameliorated tumor progression via its anti-angiogenic effects with down-regulation of vascular endothelial growth factor (VEGF) [38]. Taurine also displays a potent antineoplastic effect both *in vitro* and *in vivo* by promoting apoptosis DNA fragmentation, mitochondrial membrane loss and the release of apoptosis inducing factors [39].

Human plasma taurine levels are usually high. However, decreases were observed in response to surgery and numerous pathological conditions including cancer [40]. The anti-neoplastic effect of taurine has been the subject for many researches in the last few years. Taurine and its derivatives such as taurolidine and taurine chloranmine play a crucial role as antineoplastic agents both *in vitro* and *in vivo* through suppressing cell proliferation, enhancement of tumor cell apoptosis and inhibition of angiogenesis [41,42.]. Taurine concentrations are also high in human neutrophils and such high intracellular levels could reflect central role for taurine in modulation and maintenance the cellular functions of neutrophils. Therefore, taurine was found to modulate immune function by preserving neutrophil’s phagocytic ability that decreased due to hyperlipidemia [43]. Furthermore, taurine was reported to augment the proliferative responses of T-lymphocytes from both young and old mice; however the augmentation of the proliferative response by taurine was more marked in old than in young cells [44]. Taurine chloranmine modulates synthesis of pro-inflammatory cytokines, and therefore plays a role in the initiation and propagation of immune response [45].

Studying the immunophenotypic characteristics of peripheral blood cells of cancer patients is important. This is because the active body defenses against cancer entail the immune system. The immune system is composed of a wide range of distinct cell types with lymphocytes playing a central role providing the specificity of immune recognition [46]. CD4 T-lymphocytes have specific membrane molecules that differentiate it from CD8 lymphocytes. CD4 T-lymphocytes generally function as T- helper (T_H_) cells while CD8 T-lymphocytes function as T- cytotoxic (T_C_) cells [47].

The curcumin and taurine combination was better than single agents in treatment of human hepatoma cell propagated ex-vivo. This work revealed that a significant decrease in the cancer cell density and on contrary a significant increase in γ- interferon in cultured media [48]. Furthermore, Curcumin and taurine combination therapy of ex-vivo lymphocytes isolated from patients with cirrhosis and hepatocellular carcinoma revealed stimulation of CD4+T-helper cells with consequent induction of CD8+ cytotoxic T-cells. The latters are responsible for destroying cancer cells selectively [49,50,51,52,53].

**Side effects and Safety of the natural agents (curcumin, piperine and Taurine)**

Curcuminoids have been approved by the US Food and Drug Administration **(FDA)** as **“Generally Recognized As Safe”** (GRAS) [54). Good tolerability and safety profiles have been shown by several clinical trials.As a matter of fact, until now, clinical attempts have not discerned any adverse effects of curcumin in humans with trials using doses up to 8 grams per day[55,56].

Piperine is the major active component of black pepper and, when combined in a complex with curcumin, has been shown to increase bioavailability highly significantly [57]. Taurine (Tau) is an essential amino acid derivative in the human body, exists in various kinds of cells and exerts multiple biological actions [58]**.** Taurine has no negative side effects when used in the recommended amounts [59].

**II. Aim of the proposed study:**

- ***Primary objective:***
- Safety of CPT.
- Efficacy of CPT in halting disease progression at 6 months.
- **Secondary objective**
- Finding the best tolerated dose from a pre-specified dose range (safety).
- Other measures of treatment efficacy (response to therapy, prevention of mortality, prevention of disease progression).
- Biomarker levels: Tumor marker response (AFP, AFU levels [60]
- Immunophenotyping markers: CD8+ (cytotoxic), CD4+ (helper) T-cells and T-Reg cell (CD4+, CD25+, Foxp3+).
- Cytokines levels: Interferon Gama, TGF-Beta and IL-10.
- LDH Levels in sera of patients during the treatments.
- ***End-Point***
- **Primary:**
- Clinical benefit rate at 6 months
- **Secondary**.
- Progression free survival.
- Overall survival
- Difference in tumor marker levels (AFP and AFU) between baseline and best response.
- Difference in CD4, CD8 and T-Reg cell levels between baseline and best response.
- Difference in INF-g, TGF-B and IL-10 levels between baseline and best response.
- Difference in some specific gene expressions and Micro-RNAs

Between base-line and the best response of our applied natural agents.

**III. Methods**

**1. Trial Design**

This is a prospective two-stage non-randomized two-arm single-center phase II clinical trial.

- **The treatments:**

Oral administration of curcumin/piperine (5gm/day) devided capsules as ratio: 1000/10 mg and taurine capsules (0.5 gm/day) for three months. Repeated three cycles could be recommended.

**2. Assessment of the study treatment**

1. ***Assessment of Efficacy***

1. The efficacy parameters and methods of assessment are clinical benefit rates and response rates as measured by Progression- Free Survival (PFS), Over Survival (OS) and Response evaluation Criteria in Solid Tumors (RECIST) as measured by the Kaplan Meyer method.

2. Timing of assessments: every 3 months.

1. ***Assessment of Biomarkers***

- Assessment of tumor markers (AFP and AFU) will be using every two cycles.
- Assessment of CD4 , CD8 and T-Reg cells by flowcytometry will be done every two cycles.
- Assessment of INF-g, TGF-B and IL-10 will be done every two cycles.
- Assessment of some Micro-RNAs in sera of patients before and after treatment.
- Assessment of some specific gene expressions in leukocytes of the patient’s pre-treatment and post-treatment.

**Research Team**

Principal Investigator (1)

Motawa E. EL–Houseini Ph.D. Prof. NCI, Cairo University

E-mail: motawa_matter@yahoo.com

Mobile: 01o19013766

Home: 33869223

Principal Investigator ( 2 )

Mohamad Ali Ezz El-Arab, Hepatology consultant,National Hepatology&Tropical Medicine Research Institute(NHTMRI,Ministry of Health

E-mail: ezz2006arab@hotmail.com

Mobile: 01112233190,010052608

Research members

Mona Sayed Abdellateif

E-mail: mona.sayed@nci.cu.edu.eg

Mobile: 01140840665

Sohir Shoman, consultant,National Hepatology&Tropical Medicine Research Institute(NHTMRI),Ministry of Health

A meen Abdel Bak

Consultant at NHTMRI, Ministry of Health

Emad B.Basalious

Ph.D. Prof. Faculty of Pharmacy, Cairo University

-Shereen Saed, internal medicin,NHTMRI,Ministry of health.

- Amer Rashed, NHTMRI,Ministry of health.

**VII. References**

1. Hassan, Z.K., Hafez, M.M., Mansor, T.M., and Zekri, A.R.

HBV infection among Egyptian hepatocellular carcinoma patients. J. Virol. 2011; 8: 90.

1. Costa F. P.,deOliveira A. C.,Meirelles R.,Machado MCC. et al.

Treatment of advanced hepatocellular Carcinoma with very low levels Amplitude-modulated electromagnetic fields. British Journal of cancer. 2011; 105 : 640 – 648.

1. Luiqi Rossi,Federica Zoratto,Anseimo Papa,Francesca lodice et.al.

Current approach in the treatment of hepatocellular carcinoma Wold J.Gastrointerology Oncol. 2010; 2 (9 ):348-359.

1. Aggarwal, B.B.; Sundaram, C.; Malani, N. and Ichikawa, H.

Curcumin : the Indian solid gold. Adv. Exp. Med. Biol. 2007; 595 : 1 -75.

5 - Khopde, S. M.; Priyadarsini, K. I. and Venkatesan, P.

Free radical scavenging ability and antioxidant efficiency of curcumin and its substituted analogue. Biophys. Chem. 1999; 80-85.

1. Bengmark, S.

Curcumin, an atoxic antioxidant and natural NF Kappa B cyclooxygenase-2, lipooxygenase, and inducible nitric oxide synthase inhibitor: A shield against acute and chronic disease. JPEN .J. Parenter Enteral Nutr. 2006; 30:45.

1. Park, E. J.; Jeon, C. H.; Ko, G.; Kim, J. and Sohn, D. H.

Effect of curcumin in rat liver injury induced by carbon tetrachloride. J.Pharm. Pharmacol. 2000, 52:437-440.

1. Reddy, A. C and Lokesh, B. R.

Studies on the inhibitory effects of curcumin and eugenol on the formation of reactive oxygen species and the oxidation of ferrous iron. Mol. Cell. Biochem. 1994; 137:1.

1. Reddy, A. C and Lokesh, B. R.

Studies on spice principles as antioxidants in the inhibition of lipid peroxidation of rat liver microsomes. Mol. Cell Biochem. 1992; 111:117.

1. Wei, Q. Y.; Chen, W. F. and Zhou, B.

Inhibition of lipid peroxidaiotn and protein oxidation in rat liver mitochondria by curcumin and its analoques. Biochem. Biophys. Acta. 2006; 1760-70.

1. Dickinson, D. A.; Iles, K. E and Zhang, H.

Curcumin alters EPRE and AP-1 binding complexes and elevates glutamate-cystine ligase gene expression. FASBJ. 2003; 17:473.

1. Strimpakos, A. S. and Sharma, R.A.

Curcumin : preventive and therapeutic properties in laboratory studies and clinical trials. Antioxid. Redox. Signal. 2008; 10 :511-546.

1. Kunnumakkara A.B.; Diagaradjane, P. and Guha, S.

Curcumin sensitizes human colorectal cancer xenografts in nude mice to -radiation by targeting nuclear factor. KB-regulated gene products. Clin. Cancer Res. 2008; 14:2128-36.

1. Singh, S. and Aggarwal, B. B.

Activation of transcription factor-NF-KB is suppressed by curcumin (diferuloylmethane). J. Biol. Chem. 1995; 270:24995-25000.

1. Aggarwal, S.; Ichikawa, H.; Takada, Y.; Sandur, S. K., Shishodia, S. and Aggarwal, B. B.

Curcumin (diferulaylmethane) down-regulates expression of cell proliferation and antiapoptoic and metastatic gene products through suppression of 1KBa kinase and AKT activation. Mol. Pharmacol. 2006, 69:195-206.

1. Anand, P., Sundaram, C., Jhurani, S., Kunnumakkara, A.B., and Aggarwal, B.B

Curcumin and cancer: an "old-age" disease with an "age-old" solution. Cancer Lett. 2008; 267:133-164.

1. Sharma, R. A.; Euden, S. A.; Platton, S. L.; Cooke, D. N.; Shafayat, A. and Hewitt, H. R.

Phase I clinical trial of oral curcumin : biomarkers of systemic activity and compliance. Clin. Cancer Research. 2004, 10:6847-6854.

1. Cruz-Correa, M.; Shoskes, D. A.; Sanchez, P.; Zhao, R.; Hylind, L. M. and Wexner, S. D.

Combination treatment with curcumin and quercetin of adenomas in familial adenomatous polyposis. Clinics in Gastroentergology and Hepatology. 2006, 4:1035-1038.

1. Dhillon, N.; Aggrawal, B. B.; Newman, R. A.; Wolff, R. A.; Kunnumakkara, A. B. and Abbruzzese, I. L. Phase II trial of curcumin in patients with advanced pancreatic cancer.

Clinical Cancer Research. 2008, 14:4491-4499.

1. Bhardwaj, A.; Sethi, G.; Vadhan-Raj, S.; Bueso-Ramos, C.; Takada, Y. and Gaur, U.

Curcumin inhibits proliferation, induces apoptosis and overcomes chemoresistance through down-regulation of STAT3 and nuclear factor-kappa B-regulated antiapoptotic and cell survival gene products in human multiple myeloma cells. Blood. 2007; 109:2293-2302.

1. Anand,P.;Nair,H.B.;Sung,B.;Kunnumakkara,A.B.;Yadav,Y.R.; Tekmal, R.R. and Aggarwal, B. B.

Design of curcumin-loaded PLGA nanoparticles formulation with enhanced cellular uptake, and increased bioactivity in vitro and superior bioavailability in vivo. Biochem. Pharmacl. 2010, 79: 330-338.

1. Bishnoi M, Chopra K, Rongzhu L, Kulkarni SK.

Protective effect of curcumin and its combination with piperine (bioavailability enhancer) against haloperidol-associated neurotoxicity: cellular and neurochemical evidence. Neurotox Res. 2011;20(3):215-25.

1. Khajuria A, Zutshi U, Bedi KL

. Permeability characteristics of piperine on oral absorption--an active alkaloid from peppers and a bioavailability enhancer. Indian J Exp Biol. 1998;36(1):46-50

1. Shoba G, Joy D, Joseph T, Majid M.

Influence of piperine on the pharmacokinetics of curcumin in animals and human volunteers.

Planta Med 2008; 64(4):353-356.

24- Pantazis, P.; Varman, A.; Simpson-Durand, C.; Thorpe, J.; Ramalingan, S.; Subramaniam, D.; Houchen, C.; Ihnat, M.; Anant, S. and Ramanujam, R. P.

Tumeric and curcumin attenuate aresenic induced angiogenesis in vivo. Altern. Ther. Health Med. 2010,16(2):12-14.

25- Chuang, S. E.; Cheng, A. L.; Lin, J. K. and Kuo, M. L. Inhibition by curcumin of diethylnitrosamine-induced hepatic hyperplasia, inflammation, cellular gene products and cell-cycle related proteins in rats. Food Chem. Toxicol. 2000, 38:991-995.

26- Sreepriy, M. and Bali, G.

Effects of administration,of Embelin and Curcumin on lipid peroxidation, hepatic glutathione antioxidant defence and hematopoietic system during N-nitrosodiethylamine, Phenobarbital- induced hepatocarcinogenesis in Wistar rats. Mol. Cell. Biochem. 2006, 284:49-55.

27-Karina Reyes-Gordillo; Jose Segovia; Mineko SHibayama; Paula Vergara; Mario, G.; Mereno and Pablo Muriel.

Curcumin protects against acute liver damage in rat by inhibiting NF-KB pro inflammatory cytokines production and oxidative stress. Biochemical Biophysica. Acta. 2007, 1770: 989-996.

28- Kaur, G.; Tirkey, N.; Bharrhan, S.; Chanana, V.; Rishi, P. and Chopra, K.

Inhibition of oxidative stress and cytokine activity by curcumin in amelioration of endotoxin-induced experimental hepatoxicity in rodents. Clin. Exp. Immunol. 2006, 145:313-321.

29- Wu, S. J.; Tam, K. W.; Tsai, Y. H.; Chang, C. C. and Chao, J. C.:

Curcumin and saikosaponina a inhibit chemical-induced liver inflammation and fibrosis in rats. J. Chin. Med. 2010, 38(1):99-111.

30- Schaaf, C.; Shan, B. and Onofori, C.

Curcumin inhibits the growth , induces apoptosis and modulates the function of pituitary folliculostellate cells. Neuroendcrinology. 2010, 91(2): 200-210.

31- Huang, M. T.; Wang, Z. Y.; Georgiadis, C. A.; Laskin, J. D. and Conney, A. H.

Inhibitory effects of curcumin on tumor initiation by benzo (a) Pyrene and 7, 12 dimethylbenz (a) anthracene. Carcinogenesis. 1992, 13:2183-2186.

32- Huang, M. T.; Lou, Y. R.; Ma, W.; Newmark, H.; Reuhl, K. and Conney, A. H.

Inhibitory effects of dietary curcumin on forestomach, duodenal, and colon carcinogenesis in mice. Cancer Res. 1994, 54:5841-5847.

33- Huang, M. T.; Smart, R. C.; Wong, C. Q. and Conney, A. H.

Inhibitory effect of curcumin, chlorogenic acid, cafferic acid and ferulic acid on tumor promotion in mouse skin by 12-O-tetradecanoylphorbol-13-acetate. Cancer Res. 1988, 48:5941-5946.

34- Anand,P.;Nair,H.B.;Sung,B.;Kunnumakkara,A.B.;Yadav,Y.R.; Tekmal, R.R. and Aggarwal, B. B. Design of curcumin-loaded PLGA nanoparticles formulation with enhanced cellular uptake, and increased bioactivity in vitro and superior bioavailability in vivo. Biochem. Pharmacl. 2010, 79: 330-33.

35- Huxtable, R.

Physiological actions of taurine. Phyisol. Rev. 1992, 72: 101-163.

36- Kocaadam, B.; ¸Sanlier, N. Curcumin, an active component of turmeric (Curcuma longa), and its effects on health. Crit. Rev. Food Sci. Nutr. 2017, 57, 2889–2895.

37- Shin, H. and Linkswiler, H.

Tryptophan and methionine metabolism of adult females as affected by vitamin B6 deficiency. J. Nutr. 1974, 104: 1348-1355.

38- Calabresi, P.; Goulette, F. and Darnowski, J.

Taurolidine cytotoxic and mechanistic evaluation of a novel antineoplastiagent. Cancer Res. 2001, 61:6816-6821.

39- El Agouza I., Rawi S., and Lashin S.

Effect of taurine on rat liver injured by chronic carbon tetrachloride treatment. Egypt J. of Med Sci. 1995; 16: 185- 198.

40- Rodak, R., Kubota, H.;Ishibara, H., Eugster, H. P.,Konu, D.;Mohler, H.

Induction of reactive oxygen intermediates-dependent programmed cell death in human malignant ex vivo glioma cells and inhibition of the vascular endothelial growth factor production by taurolidine. J. Neurosurg. 2005, 102(6):1055-1068.

41- McCourt M., Fresi, Wang J., Sookai S.

Taurolidine inhibits tumor cell growth in vitro and in vivo. Annals of surgical Oncology/ 2000, 7(9): 685-691.

42- Stapleton, P.; O'Flaberty, L.; Redmond, P. and Bouchier-Hayes, J.

Host defence role for the amino acid taurine. JPEN Parenteral and Enteral Nutrition. 1998, 22:42-48.

43- Klamt, F. and Shacter, E.

Taurine chloramine, an Oxidant derived from neutrophils induces apoptosis in human B lymphoma cells through mitochondrial damage. J. Biol. Chem. 2005, 280(22):21346-21352.

44- Redmond, P.; Stapleton, P.; Neary, P. and Bouchier-Hayes, D.

Immuno-nutrition: the role of taurine. Nutrition. 1998, 14:599-604.

45- Masuda, M.; Horisaka, K. and Koeda, T.

Effect of taurine on neutrophil function hyperlipidemic rats. JPn. J. Pharmacol. 1986, 40(3):478-480.

46- Negro, S. and Hara, H.

The effect of taurine on the age-related decline of the immune response in mice, the restorative effect on the T cell proliferative response to co-stimulation with ionomycin and phorbol myristate. Adv. ExP. Med. Biol. 1992, 315:229-239.

47- Chorazy, M.; Kontay, E.; Marcinkiewicz, J. and Maslinki, W.

Taurine chloramines modulates cytokine production by human peripheral blood mononuclear cells. Amino Acids. 2002, 23(4):407-413.

48- Sasada, T.; Kimura, M.; Yashida, Y.; Kanai, M. and Takabayashi, A.):

Regulatory T cells in patients with gastrointestinal malignancies: possible involvement of regulatory T cells in disease progression. Cancer. 2003, 98:1089-1099.

49- Yang, X. H.; Yamagiwa, S.; Ichida, T.; Matsuda, Y.; Sugahara, S.; Watanabe, H.; Sato, Y.; Abo, T. Horwitz, D. A. and Aoyagi.

Increase of regulatory T-cells in the liver of patients with hepatocellular carincoma.

J Hepatol. 2006, 45 : 254-262.

50- Abd-Rabou AA.,Zoheir KM.,ahmed HH.

Potential impact of curcumin and taurine on hepatoma cells using Hu-h7 Cell line. Clin Biochem. 2012;45 (16 – 17 ) : 1519- 1521.

51- Sherif AA.Motawa E. EL-Houseini, Mamdoh El-Sherbiny Reda T., Amani S.

Ex-vivo assesment of protective effect of curcumin and taurine against Human hepatocarcinogenesis. The Journal of Basic & applied Zoology 2013 (accepted for publication).

52- Gupta, S.C.; Patchva, S.; Aggarwal, B.B.

Therapeutic Roles of Curcumin: Lessons Learned from Clinical Trials. AAPS J. 2013, 15, 195–218.

53- Vogel A, Pelletier J.

Examenchimique de la racine de Curcuma. J Pharm 1815; 1: 289-300.

54- Gupta SC, Patchva S, Koh W, Aggarwal BB.

Discovery of curcumin, a component of golden spice, and its miraculous biological activities.ClinExpPharmacolPhysiol 2012; 39: 283-99.

55- Johnson JJ, Mukhtar H.

Curcumin for chemoprevention of colon cancer. Cancer Lett 2007; 255: 170-81

56- Basnet, P.; Skalko-Basnet, N.

Curcumin: An anti-inflammatory molecule from a curry spice on the path to cancer treatment. Molecules 2011, 16, 4567–4598.

57- Hewlings SJ, Kalman DS

. Curcumin: A Review of Its’ Effects on Human Health. Foods. 2017 Oct 22;6(10):92.

58- Prentice, H.; Modi, J.P. and Wu, J.Y. (2015):

Mechanisms of neuronal protection against excitotoxicity, endoplasmic reticulum stress, and mitochondrial dysfunction in stroke and neurodegenerative diseases. Oxidative medicine and cellular longevity,2015: 1-7.

59- Rath M. Energy drinks: what is all the hype? The dangers of energy drink consumption. Journal of the American Association of Nurse Practitioners. 2012 Feb 1;24(2):70-6.

60- El-Houseini, M. E.; Mohammed, S.; Mohamed; Wael, M., El Shemey; Tarek, D.; Hussein; Omars, Desouky. and Anwar, A., El Sayed.

Enhanced detection of hepatocellular carcinoma. Cancer control. 2005; (4):248-253.
